# Evaluation of endpoints for the study and diagnosis of mitochondrial toxicity and disease: a narrative review

**DOI:** 10.1093/mutage/gead010

**Published:** 2023-05-05

**Authors:** Prashamsa Gharti, Jessica F Fletcher, Katherine E Chapman

**Affiliations:** Swansea University Medical School, Swansea University, Swansea, SA2 8PP, UK; Swansea University Medical School, Swansea University, Swansea, SA2 8PP, UK; Swansea University Medical School, Swansea University, Swansea, SA2 8PP, UK

## Abstract

Mitochondrial DNA mutation and toxicity have been linked to several inherited and acquired diseases; however, these are challenging to diagnose and characterize due to clinical and genetic heterogeneity. This review investigates current techniques for the analysis of mitochondrial perturbations, and novel, emerging endpoints for routine application within the clinical setting. Particular focus is given to the biochemistry of the mitochondria influencing each endpoint and the relation of these to toxicity. Current approaches such as the use of metabolic markers (e.g. lactate production), and muscle biopsies to measure mitochondrial proteins were found to lack specificity. Newly emerging identified endpoints were: fibroblast growth factor-21, glucose uptake, mitochondrial membrane potential, mitochondrial morphology, mtDNA heteroplasmy, and mutation of mtDNA and nuclear DNA. Owed to the advancement in genetic analysis techniques, it is suggested by this review that genotypic endpoints of mtDNA mutation and heteroplasmy show particular promise as indicators of mitochondrial disease. It is, however, acknowledged that any single endpoint in isolation offers limited information; therefore, it is recommended that analysis of several endpoints simultaneously will offer the greatest benefit in terms of disease diagnosis and study. It is hoped that this review further highlights the need for advancement in understanding mitochondrial disease.

## Introduction

Mitochondria are eukaryotic organelles specialized to carry out aerobic respiration; they function to release energy from glucose, in the form of adenosine-5ʹ-triphosphate (ATP), for cellular metabolism. Consequently, dysfunction of this organelle can disrupt many biological processes due to its role as an energy provider. Without mitochondria, energy is released inefficiently through anaerobic glycolysis with only two ATP molecules, rather than 36 molecules, produced. Mitochondrial defects are characterized by a loss of efficiency of the electron transport chain (ETC), which can lead to mitochondrial diseases (MD) (ICD10 code 88.40), a group of disorders that disrupt a range of organ systems [1]. For example, the renal proximal tubule cells and podocytes of the kidneys depend on a high concentration of ATP, and so loss of mitochondria in these cells can cause nephrotoxicity. This mitophagy-related nephrotoxicity is observed in up to 25% of patients with MD [2].

The prevalence of adult MD is high, with around 1 in 4300 individuals affected, but diagnosis is challenging due to the heterogeneous nature of MD [3]. Current diagnosis may include a metabolic approach using biomarkers, such as lactate, as an endpoint for oxidative phosphorylation (OXPHOS) defects [4]. The current gold standard in clinical detection, however, is to obtain muscle biopsies to detect morphological changes in skeletal muscle fibres [5,6]. While both have been approaches have been routinely adopted, the lack of specificity and sometimes invasive nature limits their use [5,7]. It is crucial to develop an understanding of the mitochondrial-related endpoints that can be used to identify potential toxicities in this key organelle, with a view to incorporating these into diagnostic approaches. Furthermore, it is hoped that alternative diagnostic techniques will exhibit greater sensitivity and specificity while minimizing patient discomfort associated with invasive sample retrieval. Furthermore, the incorporation of mitochondrial endpoints in clinical trials may be valuable for supporting the accurate detection of drug toxicity [8]. New methods for the detection of mitochondrial toxicity may also support the reduction and replacement of animal models [9] as they could use *in vitro* models as an alternative.

The aim of this review is to investigate the available literature discussing mitochondrial endpoints to answer three key questions; which mitochondrial endpoints are used currently in routine tests, which endpoints show promise for future use, and what evidence exists that supports their use. Here, mitochondrial endpoints are defined as biochemical indicators of mitochondrial health status, to be used as an indicator of pathology. This review will focus primarily on the biochemistry of the mitochondria that influences each endpoint, and how each can serve as an indicator of toxicity.

## Methods

A primarily narrative review approach was used to investigate and summarize the current literature within the chosen topic area. Databases used for the electronic search included PubMed, NCBI and Google Scholar, with abstracts and articles in languages other than English were excluded. The search was performed to include publications from 2015 to 2022 to provide a contemporary view. Individual research questions were subdivided into concise key search terms, including mitochondria and specific endpoints (Appendix 1). Each of these concepts was further divided into synonyms, including both general terms (e.g. ‘mitochondria’) and specific terms (e.g. ‘heteroplasmy’). Searches used Boolean operators to capture relevant papers in the field for review. When reviewing each publication, new information led to the inclusion of new key terms that could be used for further searches, as well as providing references to other related papers containing relevant information. Where other papers were found to be relevant outside of the search, these were included. Information was extracted from peer-reviewed primary research, including literature reviews and clinical trial studies. The initial search identified current diagnostic techniques used clinically to diagnose MD, followed by exploration of the underlying biochemical principles of the endpoint used.

## Mitochondrial endpoints used routinely

### Metabolic analysis using lactate

Diagnosis of patients suspected of MD is multidisciplinary, often commencing with a metabolic approach through non-invasive screening of biological samples, such as blood, to identify key metabolites relating to mitochondrial dysfunction [10]. These disease-related biomarkers reflect the presence or absence of disease, and one potentially useful ­biomarker highlighted was lactate. Dysfunctions in the ETC results in a decreased production of ATP, which can cause a switch to anaerobic glycolysis to meet the energy demand. When OXPHOS is no longer possible, pyruvate accumulates and is converted into lactate by lactate dehydrogenase, leading to the development of type B lactic acidosis [4]. An alluring benefit of using lactate as a biomarker for MD is that various samples such as cerebrospinal fluid (CSF), blood, or urine can be used, which allows for repeated measurements and is minimally or non-invasive.

Despite this, lactate levels are not always correlated to mitochondrial dysfunction. This is reiterated by a study by Bergen *et al.* investigating whether defects in mitochondrial OXPHOS complex I are linked to the development of primary open-angle glaucoma (POAG) and Leber hereditary optic neuropathy (LHON), neurodegenerative diseases distinguished by a loss of retinal ganglion cells, and are thought to involve mitochondrial dysfunction [11]. Lymphocytes were isolated and virus-transformed before being cultured in high glucose medium for 48 h and lactate levels were quantified using the media supernatant [11]. However, there was no statistically significant difference in lactate levels between the POAG control and POAG lymphoblasts [11]. This was also similarly observed in the LHON controls and LHON lymphoblasts [11]. It is important to note that the data showed no statistical significance for POAG (*P* = 0.25) and LHON (*P* = 0.63) [9]. Lactate concentration may differ depending on when the sample was taken; if taken during ­exercise then ­lactate levels will naturally increase [11]. Another possible limitation of this study is that modelling using peripheral tissues such as lymphoblasts may mean that some pathological features are not observed in those models [11], although it is acknowledged that lymphoblastoid models can aid the study of mitochondrial disorders [12–19]. This study therefore highlights the limitations of measuring lactate as a mitochondrial endpoint.

### Muscle biopsy

Skeletal muscle biopsy is another routine diagnostic method that has been used for many years, being considered the gold standard. It can be used to detect mitochondrial myopathies, a common manifestation of mitochondrial dysfunction affecting OXPHOS due to the high energy demand of skeletal muscles [5]. There are important morphological alterations that implicate MD and can be detected through staining of muscle cryosections: ragged red fibres (RRF), succinate dehydrogenase (SDH) reactive blood vessels, and cytochrome C oxidase (COX)-negative fibres [6]. Modified Gomori trichome staining highlights connective tissue, muscle fibres, and mitochondria, which reveals the presence of RRF [11]. RRF exhibit a ‘fibre cracking’ appearance and arise from mitochondrial aggregation in the subsarcolemmal region of the fibre to compensate for OXPHOS deficiency [11]. Modified Gomori trichome staining highlights aggregates in red, and the periphery perimeter of the muscle fibre in blue. Similarly, biopsies of SDH blood vessels contained proliferated mitochondria aggregates. Typical muscle biopsy diagnosis is through sequential COX/SDH histochemistry; activities of SDH (complex I) and COX (complex IV) are collated and analysed through staining on a slide. A mosaic of COX-negative fibres, signalling low COX activity, with reduced SDH activity are observed as blue compared to normal COX-positive fibres which appear as brown. This is caused by mutations in the mitochondrial DNA (mtDNA), creating different levels of mutational heteroplasmy thus forming a spectrum of positive and negative fibres [5, 11]. mtDNA contains a total of 37 genes which code for 13 proteins of OXPHOS complexes, 22 transfer RNAs (tRNAs), and two ribosomal RNAs (rRNAs), therefore mutations can disrupt OXPHOS complexes production leading to the morphological changes observed [20].

The studies acknowledge a crucial weakness that stems from a lack of specificity; muscle biopsies can appear normal in some patients and RRFs or COX-negative fibres can occur naturally from normal ageing processes or due to non-mitochondrial related muscle disorders. However, some studies introduce a threshold that must be met to confirm a diagnosis of mitochondrial myopathy; for example, patients over 50 years of age presenting with COX-negative fibres at a frequency of >5% or in patients 30–50 years a frequency of >2% or for patients of any age and RRF frequency of >2% could be considered a positive diagnosis for MD [5, 6, 21]. Despite this, an absence of these fibres does not completely exclude a diagnosis of MD [11]. The biopsy may fail to detect subtle OXPHOS weaknesses when only a few muscle fibres are detected, for example, children under five typically present with only mild subsarcolemmal mitochondrial aggregation [11]. Further to these issues, a large mass of muscle ­tissue is required for testing (50–100 mg), and so the extraction process is invasive for the patient. Overall, while the morphological changes of specific muscle fibres can in some cases indicate mitochondrial toxicity, this method is insufficient in isolation due to lack of specificity and must be combined with other methods.

## Promising mitochondrial endpoints: phenotypic endpoints

### Fibroblast growth factor-21

Fibroblast growth factor-21 (FGF-21) is a circulating hormone and shows improved potential as a mitochondrial endpoint over conventional serum diagnostic tests [22]. Although the function of FGF-21 is yet to be fully understood, recent studies revealed that patients with ETC deficiencies show an increased myocytic expression of FGF-21 [23–27]. Morovat *et al*. [27]. investigated implementing FGF-21 measurements as a first-line laboratory test, in hopes of reducing the need for muscle biopsy in diagnosis. Investigating 155 patients, a diagnosis of MD was established in 104 based on DNA analysis [27]. Z-scores were used to quantify the standard deviation above or below the mean and z-scores established as ≥2 were defined as clinically significant. FGF-21 concentrations were observed to be higher in patients with MD, and highest in those who had mtDNA maintenance defects (*n* = 32) and rearrangement defects (*n* = 17) (median FGF-21 z-score for both groups = 1.99) [27]. On the contrary, patients who had MD, due to mtDNA point mutations or other autosomal gene mutations, and patients where a definitive MD diagnosis could not be made, exhibited lower FGF-21 concentrations [27]. These results suggest that measurement of FGF-21 cannot be entirely useful on its own, only proving to aid diagnosis in around one-third of patients [27] investigated for MD. Despite this, the authors remain optimistic that serum FGF-21 measurement can improve diagnosis by replacing the use of muscle biopsies, and could be used to triage patients suspected of MD for genetic analysis confirmation. It also reveals that this method has a positive predictivity above 90%, which improves upon the low positive predictivity of current first-line diagnostics.

However, there were inconsistencies in arguments supporting FGF-21 measurements [27]. Finsterer and Zarrouk-Mahjoub [22] highlight that FGF-21 is primarily produced in skeletal muscle, which contrasts with the general notion that the liver and adipose tissue are the primary source of FGF-21. Working from the assumption that skeletal muscle is a primary source, FGF-21 may only be a useful mitochondrial endpoint for patients with myopathies as normal levels have been observed in MD that do not manifest as myopathy. Other analyses of FGF-21 concentrations further support an elevation of the biomarker only in correlation to mitochondrial deficiencies manifesting in muscle defects [28]. The high specificity noted is also contrasted by this literature, stating that although serum concentrations are elevated in patients with MD, specificity is low because increased levels have been observed in other pathologies such as obesity and in response to stress [29].

### Glucose uptake/mitochondrial membrane potential using tracers

Imaging glucose uptake and mitochondrial membrane potential (ΔΨm) in the tissue microenvironment as mitochondrial endpoints is a novel approach [30]. The Warburg effect describes how most cancers exhibit an increased dependence on glycolysis to meet the increased energy demands needed for cancer progression. Previous data have highlighted this dependency as cancer growth is impaired when OXPHOS is reduced [31]. Cancers unusually convert glucose to lactate despite there often being sufficient oxygen present for cells to respire aerobically [32]; this leads to the conclusion that in cancer, mitochondria may be defective. This is evident in studies where inhibiting lactose dehydrogenase, thus preventing the conversion of pyruvate to lactate, reduces oncogenesis. ΔΨm occurs due to the activity of proton pumps in the ETC and this factor serves as a mitochondrial endpoint predictive of mitochondrial viability [33]; higher ΔΨm means cells are more susceptible to forming tumours [33, 34].

There are currently many methods that facilitate imaging these metabolic axes at an organ-level, such as Positron Emission Tomography using fluorodeoxyglucose as a tracer for glucose uptake. Zhu *et al*. [30]. outlined a need for new metabolic approaches to combine these *in vitro* and *in vivo* analyses to improve resolution and provide better quality information. Animal models’ systemic properties confer advantages when studying mitochondrial effects. Two indicators were used in the study: 2-[*N*-(7-nitrobenz-2-oxa-1,3-diazol-4-yl) amino]-2-deoxy-d-glucose (2-NBDG) and Tetramethylrhodamine ethyl ester (TMRE), which detect glucose uptake and mitochondrial membrane potential respectively. The delivery scheme features a TMRE injection followed by a 2-NBDG injection after a 10–15 min delay [30]. This eliminates the inhibition of TMRE uptake by simultaneous injection of 2-NBDG; the study revealed that when both fluorophores are injected, TMRE fluorescence is lightly attenuated by the presence of 2-NBDG [30]. When imaging murine breast cancer models, TMRE intensity decreased more under hypoxic conditions (10% oxygen) than normoxia (21% oxygen) [30]. On the other hand, 2-NBDG increased under hypoxia compared to normoxia [30]. Previous data demonstrated that glucose uptake usually increases in hypoxic conditions [30]. Both TMRE and 2-NBDG intensity increased in 4T1 tumours compared to non-tumour tissues [30]. It is hypothesized that as tumours develop and create hypoxic regions, it will shift towards increased glucose uptake. Coupled with optical technology, this fluorescence imaging technique provides the potential for metabolic studies for many diseases relating to mitochondrial toxicity, including cancer, as well as providing benefits of high resolution and repeatability.

However, due to advancements in technology for screening mitochondria, it was discovered that cancer cells still rely on functional mitochondria; the Warburg effect is not observed in all cancer cell types [35]. One study explored the reliance on mitochondria by deleting mtDNA in metastatic murine B16 melanoma and 4T1 breast carcinoma cells, producing B16p^0^ and 4T1p^0^ cell lines, respectively [36]. When these cell lines were injected into mice intravenously, both B16p^0^ and 4T1p^0^ cells failed to develop tumours in the lung initially, while the parental cell lines (B16p and 4T1p, containing normal mtDNA) did undergo tumorigenesis. B16p^0^ and 4T1p^0^ could only form tumours after acquiring host mtDNA [36]. Mitochondria assist cancer progression by altering their bioenergetics and providing building blocks for the formation of the tumour cells [36]. Some cancers rely on mitochondrial toxicity as it affects OXPHOS but, for cancers that rely on mitochondrial function, glucose uptake and mitochondrial membrane potential may not serve as an effective diagnostic endpoint [36]. In these situations, other mitochondrial endpoints may be more appropriate.

### Mitochondrial fission and fusion

Mitochondrial morphology changes when influenced by cellular environmental stimuli, such as nutrient stress, and is associated with their ability to efficiently release energy. The literature demonstrates that mitochondrial morphology may be associated with several diseases, with mitochondrial fission and fusion processes contribute towards altered mitochondrial morphology [37].

A balance between mitochondrial fission and fusion is needed to maintain mitochondrial function; for example, cells exhibiting more fission generate short/small rod mitochondria (fragmented) [38]. Fragmented morphology is linked to metabolic dysfunction and disease, and mitochondrial abnormalities may be associated with disruption to cellular communication and homeostasis [39]. Drp1, for example, is the main regulator of mitochondrial fission, with its activation disrupting mitochondrial homeostasis which promotes oxidative stress through increased ROS production [38]. ROS causes cellular damage and correlates to mitochondrial fragmentation. Cancer cells exhibit an imbalance between fission and fusion processes and there is potential for Drp1 to be used to predict prognosis and treatment response or as a therapeutic target in cancer and other diseases [38, 40, 41]. Human lung cancer cells show imbalance of homeostasis (more fission than fusion); this disease phenotype can be reversed by Drp1 inhibition or Mfn2 overexpression, another mitochondrial morphology regulator [38]. There are several important cellular pathways that contribute towards regulating mitochondrial fission and fusion, including the mitogen-activated protein kinase and ubiquitination [40].

## Promising mitochondrial endpoints: genotypic endpoints

### Mitochondrial heteroplasmy

With mitochondrial heterogeneity remaining the main challenge of diagnosis, being able to quantify mutations in mtDNA could be important for improving diagnosis. Currently, analysing homoplasmic mtDNA mutations is uncomplicated using simple laboratory techniques. However, since each cell contains many mitochondria with thousands of copies of mtDNA, wild-type and mutant mtDNA can coexist in a single cell, known as heteroplasmy. Furthermore, during cell division, mitochondria are randomly distributed into daughter cells, further creating a varying level of heteroplasmy [42]. For example, mutations of ND5 protein, a subunit for NADH dehydrogenase, create different disease phenotypes depending on this variability; homoplasmic mutations inhibit tumour growth but heteroplasmic mutations promote growth [43]. This suggests that heteroplasmy could be applied as a marker for cancer progression.

The mitochondrial genome is more vulnerable to mutations when compared to the nuclear genome; mtDNA mutation rate exceeds that of nuclear DNA by at least 10-fold [44, 45]. There are more than 200 mtDNA point mutations that have been recorded, located in all mitochondrial genome regions, and this number continues to increase with further research. This is likely due to a lack of genome protection, such as a limited DNA repair system, a lack of histone protein protection seen in nuclear DNA, and a lack of introns. Therefore, any mutations will affect coding sequences and therefore likely exhibit biological consequences.

Mitrofanov *et al*. [43]. linked mitochondrial toxicity to the development of atherosclerosis, and aimed to develop a procedure to permit quantification of heteroplasmy to identify risk for nine mtDNA mutations associated with the disease: A1555G, C3256T, T3336C, C5178A, G12315A, G13513A, G14459A, G14846A, and G15059A. DNA was isolated from 325 individuals, composed of those categorized as healthy participants (*n* = 100, group 1), patients with ultrasound signs of carotid atherosclerosis (*n* = 125, group 2), and ischaemic heart disease (IHD) (*n* = 100, group 3) [43]. Using real-time polymerase chain reaction (PCR) assays, the accumulation of signal from a fluorescent dye attached to a probe complementary to the mtDNA sequence was recorded [43]. Artificial oligonucleotides recreating the mtDNA with the mutant allele were used, each containing different heteroplasmic mixtures (100, 93.7, 87.5, 75, 50, 33, 25, 12.5, and 6% mutant allele), with mutant allele population determined by analysing the probe fluorescence curves [43]. The method was successful in detecting heteroplasmy in the participants; group 3, composed of patients with IHD, showed the highest average heteroplasmy for mutations C3256T (13.9%) and G12315A (14.8%) [43]. This was smaller in mutation G13513A for group 3, compared to groups 1 and 2 (12% vs 20% and 16%, respectively), because the mutant allele overcame any non-mutant alleles in the healthy participants [43].

Historically, mtDNA detection has been performed using Southern blotting, although this is restricted by its requirement of large amounts of DNA sample. PCR can overcome this issue as it permits amplification of low starting quantities, possibly single molecules [46, 47]. Furthermore, the study outlines the challenge of heteroplasmy, noting that it can vary substantially between different tissues due to their different energy demands. DNA samples extracted from different sources, such as blood and urine, can show contrasting results for this method, and therefore a negative result generated does not eliminate the presence of mtDNA mutations [47]. Furthermore, heteroplasmy levels can vary substantially between cells, tissues, and family members, and be influenced by age and other factors [48]; this may therefore mean that it is a weak and unreliable indicator of mitochondrial toxicity. Refinement of this methodology is necessary, and there is still a need to combine this genetic testing with tissue biopsies to provide a conclusive diagnosis of mitochondrial toxicity or disease.

### General mitochondrial DNA mutation

Characterization of mtDNA variants has the potential for improving understanding and diagnosis of disease. Next Generation Sequencing approaches represent a major advancement in genetic analysis and may be applied to the mitochondrial genome. At ~16.6 kb in length, sequencing of the whole mitochondrial genome is becoming more affordable, and perhaps is a more practical option than sequencing the entire nuclear genome. mtDNA sequencing is already contributing to improved diagnosis of patients with inherited mitochondrial disease exhibiting homoplasmy [49]. Given that mitochondria are maternally inherited, women with pathogenetic mtDNA mutations may now have the option for mitochondrial donation as part of *In Vitro* Fertilization [49]. In diseases such as cancer, the issue of mtDNA mutations occurring at low frequency may make detection of specific mtDNA mutations driving the disease challenging; a possible solution is the use of more advanced sequencing approaches such as duplex sequencing. Another method showing ­promise is droplet digital PCR (ddPCR), which may allow quantification of absolute numbers of mtDNA copies at both the cell population and single-cell level, due to increased precision compared to traditional PCR methodologies [50, 51]. To date, measuring mtDNA copy number has been challenging and has limited diagnostic accuracy [50].

A potential limitation of mtDNA sequencing and copy number data viewed in isolation is the specificity; individual mtDNA variants’ impact, if any, on phenotype and/or disease state may be unclear based on sequencing outputs alone [52]. With a high frequency of benign variants naturally occurring within mtDNA, identification of pathogenic variants is challenging [52]. It is likely that studying multiple datasets ­simultaneously would allow pathogenic variants to be identified with greater ease; for example, in addition to obtaining mtDNA sequence data, the mitochondrial transcriptome, nuclear genome data, and mitochondrial metabolomics could also be assessed [52]. This broader, multi-omics approach would allow for a more informative, integrated approach. With such large datasets, systematic documentation and open access to repositories for mtDNA mutation data are crucial, such as the Mitochondrial Disease Sequence Data Resource (MSeqDR) [53].

### Nuclear DNA genes associated with mitochondrial function

While the mitochondrial genome encodes 13 mitochondrial proteins, the mitochondrial proteome consists of approximately 1500 different proteins [54]. The vast majority of proteins contained within the mitochondria are therefore encoded by the nuclear genome rather than the mitochondrial genome, with nuclear genes contributing not only to respiratory chain subunits but also mitochondrial maintenance; for example, the nuclear gene *POLG* encodes the DNA polymerase gamma subunit employed in the mitochondria [54]. While nuclear gene mutation analysis is not a mitochondrial endpoint *per se*, mutation in these genes is known to contribute to MD [54–56]. For example, mutations in relevant nuclear genes can lead to mtDNA instability and MD [54]. There are a variety of methodologies available to measure nuclear DNA mutation, including Next Generation Sequencing; description of these lies beyond the scope of this mitochondria-focused review. Nevertheless, it is acknowledged that the potential role that nuclear gene mutations play in the development and diagnosis of MD is important.

## Discussion

Mitochondrial endpoints may be useful for the understanding and diagnosis of certain diseases. Today, the main challenge associated with detecting mitochondrial toxicity *in vivo* originates from the multi-systemic nature, due to the different energy demands of each organ system [57]. Moreover, different disease phenotypes might be caused by mitochondrial heteroplasmy, where the cell population exhibits variable levels of mutant and wild-type mtDNA [3, 46]. Consequently, there has been a pursuit of promising mitochondrial endpoints for incorporation as diagnostic and research tools. Table 1 summarizes the projected advantages and limitations of the identified emerging endpoints.

**Table 1.** Summary of emerging mitochondrial endpoints’ advantages and limitations.

Sample type	Endpoint	Advantages	Limitations
Phenotypic	FGF-21	High positive predictivity (>90%) and specificity for MD	May only be useful for MD patients with myopathies
	Glucose uptake/mitochondrial membrane potential using tracers	High resolution and repeatability	May not detect diverse range of cancer phenotypes
	Mitochondrial fission and fusion	Associated with several diseases (e.g. Drp1 changes), with relation to cellular function	Incomplete understanding of how morphology links to disease Other factors may affect mitochondrial morphology; inter-individual variation
Genotypic	Mitochondrial heteroplasmy	Useful for characterizing possible sources of MD in terms of mutation profile	May vary substantially between tissues and individuals; may be difficult to quantify accurately within the sample sample
	Mitochondrial DNA mutation	Rich datasets, possible to single cell/sequence level Low mass of sample can be used	Low specificity Knowledge of heteroplasmy required Some homology to nuclear genes
	Nuclear DNA genes associated with mitochondrial function	Useful for characterising type of MD in terms of mutation profile Low mass of sample can be used	Functional implications of mutations not always clear

FGF-21 is promising for the replacement of muscle biopsies in diagnosing MD related to muscle disorders [27]. Using tracers, glucose uptake and mitochondrial membrane potential have been successfully detected in cancers with ­mitochondrial dysfunction [29]. Yet, this is not suitable for tumours where mitochondria are functional [42], indicating that other endpoints need to be combined to form a conclusive diagnosis. This is similarly seen in quantifying mitochondrial heteroplasmy; heteroplasmy variability in different tissue samples means that the absence of mutated mtDNA cannot be eliminated [57] and will need to be coupled with results from tissue biopsies. Despite this, analysis of heteroplasmy seems to be a promising mitochondrial endpoint due to its specificity and could replace current Southern blot testing [47]. mtDNA sequencing is similarly showing promise in this area, although a multi-omics approach is recommended to ensure that pathogenic mtDNA variants can be correctly identified [52].

Other mitochondrial endpoints used in research may have the potential for expanded use in the clinic, such as markers linked to mitochondrial functionality. These include ROS production, ATP production, oxygen consumption, and glucose/galactose assays. For example, respiratory screening technology (RST) and the Seahorse Analyzer may be applied to measure oxygen consumption as a marker of mitochondrial respiration in both *in vitro* and, crucially for MD diagnosis, *ex vivo* samples [8]. In particular, RST has shown reasonable predictive capacity for drug hepatotoxicity, with possible applications in early drug discovery processes [8]. Measuring the production of mitochondrial ROS (mtROS) can indicate overall mitochondrial health status and functionality. mtROS is primarily produced at complexes I and III of ETC when electrons from NADH or FADH2 react with oxygen [58]. This leads to ROS over-production, causing oxidative damage to mitochondrial components such as proteins, lipids and DNA, in turn disrupting ETC functionality [58]. Investigation of the role of mtROS on changes in mitochondrial function (as well as other functions) in mouse models that mimic pathology of IRI-AKI (ischaemia reperfusion injury-acute kidney injury) concluded that mtROS promotes renal injury [58]. There are several cellular sources of ROS, as such species are produced as a byproduct of endogenous cellular processes; notable sources include peroxisomes [59], xanthine oxidase [60], and the endoplasmic reticulum [61].

While functional analysis can offer insights into mitochondrial health, these may have limited benefit for diagnosis of mitochondrial disease as again they lack specificity and were therefore not considered to be endpoints of greater promise within this review [62].

Based on this review, future studies should focus on refining high-throughput genetic approaches, such as quantifying mitochondrial heteroplasmy levels and mtDNA mutation. A combined, multiple-endpoint approach may be beneficial at this juncture, to allow the correct targeting of gene variants that are directly associated with mitochondrial disease. There may then be potential for the development of a method that does not require a need for other mitochondrial endpoints to provide conclusive diagnosis and overcomes the variability in different sample types and invasive sampling.

## Appendix 1: List of example search terms used for database searches

1. (mitochondri* AND (endpoint* OR biomarker* OR toxicit* OR dysfunction OR defect OR parameter))mtDNA OR mitochondri* DNA OR mitochondri* genome
2. Mitophagy OR mitoxicity
3. Measur* OR detect* OR quantif* AND mitochondri*
4. #1 AND morphology
5. Cancer AND mitochondria
6. Mitochondri* disease OR myopath* OR disorder*
7. #1 AND FGF-21 OR fibroblast growth factor-21*
8. #4 AND heteroplasmy
9. Mitochondri* diagnosis OR screening OR test*
#10 AND muscle biopsy OR screening
#11 AND Gomori trichrome
Measur* lactate
#1 AND aerobic OR anaerobic respiration OR glycolysis
#1 AND ETC OR ETC OR respiratory chain
#1 AND membrane potential
Warburg effect AND mitochondri*
